# P-494. The Silver Tsunami Is Upon Us: Over Half of Persons with HIV in the United States Are Aged ≥ 50 Years and Almost Half Have Been Living with Diagnosed HIV for ≥ 15 Years

**DOI:** 10.1093/ofid/ofae631.693

**Published:** 2025-01-29

**Authors:** Siobhan M O’Connor, Alexandra B Balaji, Scott Grytdal, Baohua Wu, Xiaohong Hu, John Brooks

**Affiliations:** Centers for Disease Control and Prevention, Atlanta, GA; Centers for Disease Control and Prevention, Atlanta, GA; Centers for Disease Control and Prevention, Atlanta, GA; Centers for Disease Control and Prevention, Atlanta, GA; Centers for Disease Control and Prevention, Atlanta, GA; Centers for Disease Control and Prevention, Atlanta, GA

## Abstract

**Background:**

On average, persons with diagnosed HIV (PWH) are living longer, and PWH who are engaged in effective care can expect lifespans comparable to persons without HIV. Understanding changes in the age structure of PWH can help plan where to direct healthcare resources to match need, especially medical needs related to aging and to preventive care for AIDS- and non-AIDS-related comorbidities.

**Figure:**
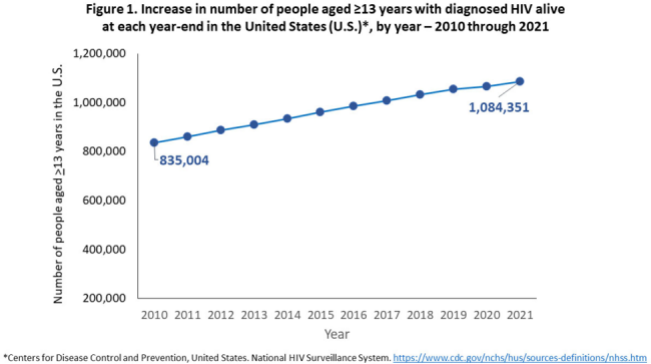

**Methods:**

Using data from CDC’s National HIV Surveillance System (NHSS) for PWH aged ≥ 13 years in the United States, we compared changes in the number of PWH alive at each year-end over the period 2010-2021, examined changes in their age structure, and for 2021 calculated duration of HIV infection since diagnosis for all PWH aged ≥ 18 years.

**Figure:**
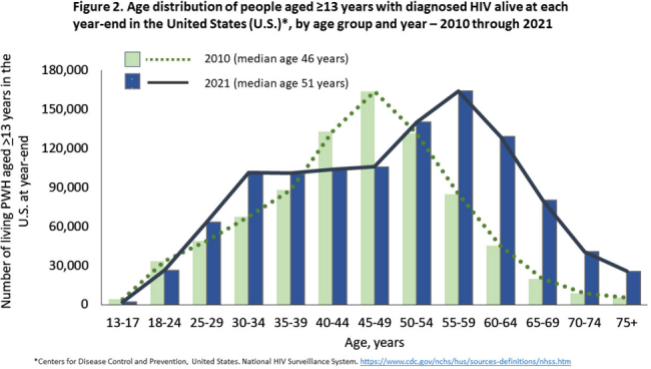

**Results:**

During 2010-2021, the overall number of PWH increased 29.9%, from 835,004 in 2010 to 1,084,351 in 2021 (Fig 1). The number of PWH aged ≥ 50 years increased 96.3%, from 295,464 to 580,011, and the number aged ≥ 65 years increased 335%, from 33,646 to 146,485 (Fig 2). At year-end 2021, overall median age was 51 years, 53.5% of all PWH were aged ≥ 50 years, and 13.5% were aged ≥ 65 years. Of all adult PWH, 45.8% (497,005) had lived with HIV over 15 years since diagnosis. Among the subset of 12,563 PWH living with perinatally acquired HIV, the median age at year-end 2021 was 26 years; 31.4% were infected at least 30 years, and 76.6% at least 20 years.

**Conclusion:**

From 2010-2021, the number of PWH aged ≥ 50 years increased markedly and overall median age of PWH aged ≥ 13 years increased to exceed 50 years; 1 in 7 were aged ≥ 65 years. By year-end 2021, PWH aged ≥ 50 years totaled over one half million, among whom 1 in 4 was aged ≥ 65 years. Almost half of adult PWH had lived with diagnosed HIV for at least 15 years and three-quarters of young adults with perinatally acquired HIV had lived with HIV for 20 years or more. Our findings highlight the large and rapidly growing need not only for expertise treating older PWH but also integration of prevention strategies to minimize the impact of AIDS- and non-AIDS-related comorbidities of aging in PWH with long-standing HIV.

**Disclosures:**

**All Authors**: No reported disclosures

